# Neonatal Subcutaneous Emphysema Secondary to Chest Tube Placement Using the Trocar Technique: A Case Report

**DOI:** 10.7759/cureus.51879

**Published:** 2024-01-08

**Authors:** Taha S Jilani, Jocelin Loewen, Seham Azzam, Samer Bou Karroum, Olubukunola A Adesanya

**Affiliations:** 1 Pediatrics, Texas Tech University Health Sciences Center, Amarillo, USA

**Keywords:** neonatal intensive care unit (nicu), thoracostomy tube, pneumothorax, pigtail, trocar, chest tube, neonate, subcutaneous emphysema

## Abstract

Subcutaneous emphysema is a type of air leak in which air accumulates within the subcutaneous layer of the skin underneath the dermal layers. The accumulation of air can be seen on imaging in relevant body areas such as the abdomen, chest, face, or neck. During physical examination, crepitus, the sensation or sound of crackling upon palpation, is the most common associated finding. Various causes for subcutaneous emphysema exist, with one such cause being thoracostomy or chest tube placement. The trocar technique, in particular, has been associated with greater complications when compared to other techniques. Here, we present a case of subcutaneous emphysema in a neonate occurring after placement of a chest tube using the trocar technique. At this time, much of the knowledge regarding subcutaneous emphysema related to chest tube placement is in the adult population. Clinicians should be aware of this complication in neonates as the body of knowledge regarding this topic continues to grow.

## Introduction

Subcutaneous emphysema (SE) refers to a type of air leak in which there is a presence of air within the subcutaneous layer of the skin [1]. SE occurs when gas or air accumulates and seeps under the skin, where gas should not be present. Subcutaneous refers to the subcutaneous tissue, and emphysema refers to trapped air pockets. As the air generally comes from the chest cavity, SE usually occurs around the upper torso, such as on the chest, neck, face, axillae, and arms, where it can travel with little resistance along the loose connective tissue within the superficial fascia, In most cases, the air leak manifests as visible distension or bloating in the chest, abdomen, neck, or face. The most common notable finding is crepitus, a characteristic sound and sensation of crackling upon palpation of the affected areas [1]. Common causes of SE include tissue infections, blunt or penetrating trauma, intubation, and iatrogenic [2]. In hospitalized patients, SE commonly occurs because of pneumothorax or pneumomediastinum [2]. Imaging such as chest X-ray (CXR) and chest computed tomography (CT) scans can aid in diagnosis. On CXR, intermittent radiolucent areas often appear as areas of fluffiness on the exterior borders of the thoracic walls. More specifically, there can be striations of gas along the pectoralis major, a finding known as the ginkgo leaf sign [1]. CT scans show gas in the subcutaneous layer, demonstrated as dark pockets. Although CXRs are often the most immediate diagnostic tool, CT may be more sensitive in determining the source of injury [1].

While the presentation of SE is well-documented in the adult population with signs and management clearly defined, it is a rarely documented occurrence in the neonatal population [3]. In neonates, SE is associated with pneumothoraces or pneumomediastina, either as secondary presentations or consequence of treatment for these conditions [3]. Both pneumothoraces and pneumomediastina are often treated conservatively. However, thoracostomy, or chest tube placement, is commonly utilized when conservative management fails or when surgical drainage is required for treatment. Other indications for chest tube placement include the removal of abnormal fluid accumulation. Examples include pleural effusions, hemothoraces, empyemas, and chylothoraces [4].

When chest drainage is required, options for tube placement include traditional chest tube placement using a trocar with dissection or using a Seldinger technique with a pigtail catheter. As the name suggests, the trocar technique utilizes a rigid tip trocar to insert the chest tube through the outside tissue and intercostal muscle layers and into the pleural cavity. While all methods are invasive and can result in technical or infectious complications, studies have shown that the trocar technique carries a higher risk of complications and often leads to more intrathoracic or extrathoracic injuries compared to other methods [4]. However, almost all these documented cases are in the adult population with little data regarding neonates. Here, we present a case of SE in a neonate occurring after the placement of a chest tube using the trocar technique.

## Case presentation

A late-term, 41+1 weeks, appropriate for gestational age male newborn weighing about 4 kg was delivered via cesarean section due to arrest of cervical dilation and failure to progress to a 22-year-old gravida 1 para 1 with adequate prenatal care. The mother was rubella equivocal and group B *Streptococcus* positive with adequate treatment, with all other serologies being negative. The membrane was ruptured artificially about nine hours before delivery with clear amniotic fluid. APGAR scores were 8/8 at one and five minutes, and the newborn examination showed a normal neonate in no acute cardiopulmonary distress.

About four hours after birth, the neonate began to desaturate in the nursery with oxygen saturations ranging from 85% to 92% on continuous oxygen saturation monitoring with a difference in pre- and post-ductal saturations of about 3-5 points. During episodes of crying, desaturation was more prominent with levels reaching 70s on room air but otherwise stable vitals. On the physical examination, the neonate was pale, capillary refill time was about four to five seconds, and heart sounds were faint on the left and more pronounced on the right.

**Figure 1:**
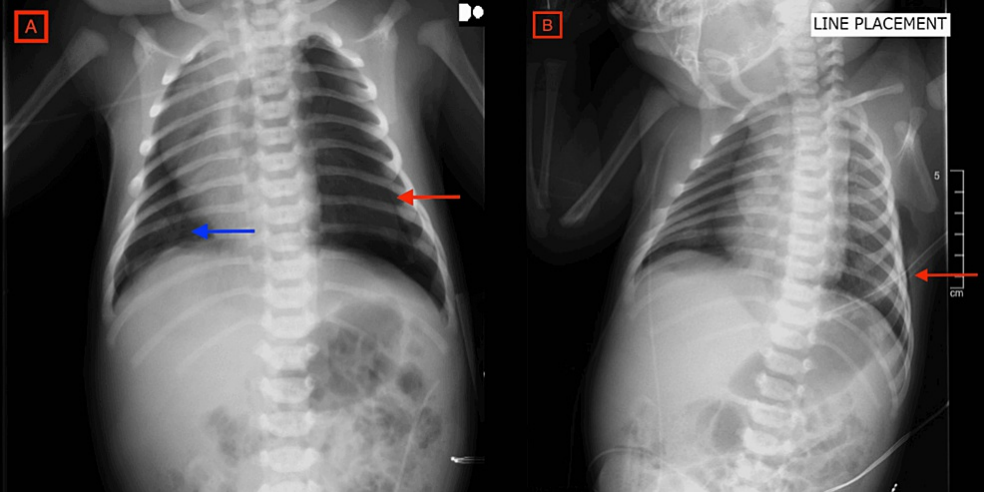
A. Chest X-ray at day one of life documenting the presence of large left-sided tension pneumothorax before tube placement (red arrow). There is a moderate-sized right pneumothorax which is partially obscured by the mediastinal shift (blue arrow). B. Chest X-ray on the first day of life after left chest tube placement (red arrow) without signs of subcutaneous emphysema.

Single film CXR showed a small, right-sided pneumothorax, a depressed left hemidiaphragm, a right-sided shift of mediastinum, and a developing left-sided tension pneumothorax. With a recommendation from the reading radiologist, a repeat four-view CXR was ordered and showed moderate-sized right pneumothorax, mild right basilar opacity suggestive of atelectasis, and a large left-sided pneumothorax that had worsened since initial imaging with signs of tension pneumothorax (Figure 1A). An echocardiogram just before the repeat four-view CXR revealed a patent foramen ovale and a small patent ductus arteriosus but was otherwise normal. Stat chest tube placement was recommended by radiology and thus the neonate was transferred immediately to the neonatal intensive care unit (NICU) down the hall and placed on a 1 L high-flow nasal cannula (HFNC). In the NICU, a 10 French Trocar thoracostomy was performed at the left midline. The tube was placed on continuous suction with bursts of air and serosanguineous fluid. Post-procedural CXR confirmed proper chest tube placement and slightly increased right-sided pneumothorax (Figure 1B). At this time, breath sounds were noted to be decreased in the left upper area.

**Figure 2:**
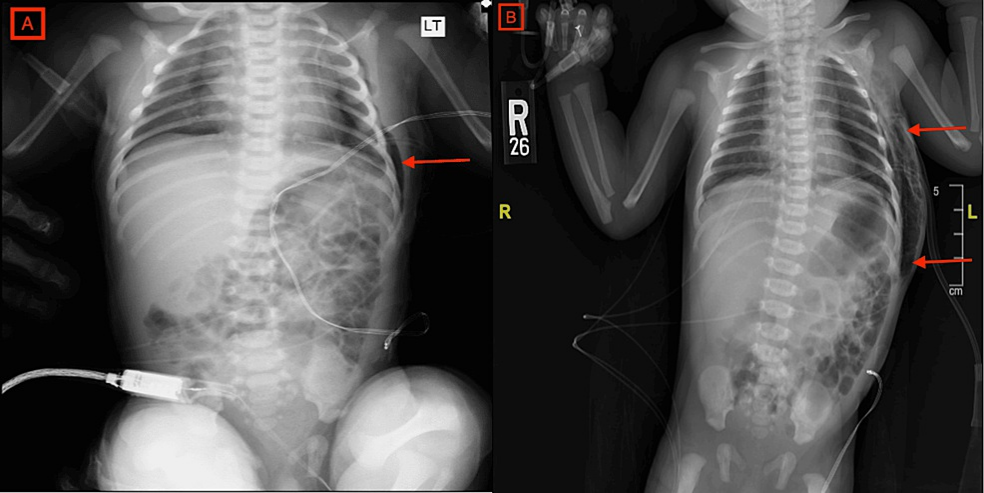
A. Chest X-ray on the first day of life showing initial left-sided subcutaneous emphysema (red arrow). B. Chest X-ray on the third day of life showing left-sided subcutaneous emphysema increased in size from previous imaging, tracking upper chest and abdomen anteriorly and posteriorly (red arrows).

Approximately 10 hours after tube placement, repeat two-view CXR showed signs of left-sided subcutaneous soft tissue emphysema on the left chest wall (Figure 2, Panel A). Up to this point, the neonate had intermittent tachypnea with respirations in the 60s-80s and mild retractions but was otherwise stable with oxygen saturations ranging from 91% to 100% on 1 L HFNC with a fraction of inspired oxygen (FiO_2_) of 21-40%. Complete blood count, comprehensive metabolic panel, and capillary blood glucose were all within normal limits.

On his second day, the neonate presented with respiratory distress most likely secondary to spontaneous pneumothoraces and transient tachypnea of the newborn. Repeat CXR showed left-sided SE, a decrease in the size of pneumomediastinum, and small bilateral pneumothoraces. Chest tube suction was turned off and the neonate was weaned off HFNC to room air.

On his third day, repeat CXR showed small right and left basilar pneumothoraces unchanged from before and an increase in the size of the left-sided SE on the chest wall. The emphysema was found to be larger and tracked to the upper chest and abdomen anteriorly and posteriorly (Figure 2, Panel B). At this time, the neonate’s respiratory distress was resolving with mild retractions and stable oxygen saturations of 92-100% on room air.

Repeat CXR on day four showed a decrease in the size of the left pneumothorax while the right remained unchanged. The large SE was noted to be slightly decreasing. The chest tube was removed as the patient was clinically improving. Repeat CXR on day five showed that the SE was still present on the left chest wall and upper abdomen and unchanged from prior imaging. On examination, significant crepitations along the entire chest, back, and abdomen were still appreciated. However, no respiratory distress or increased work of breathing was noted, and the neonate was still deemed clinically stable.

**Figure 3:**
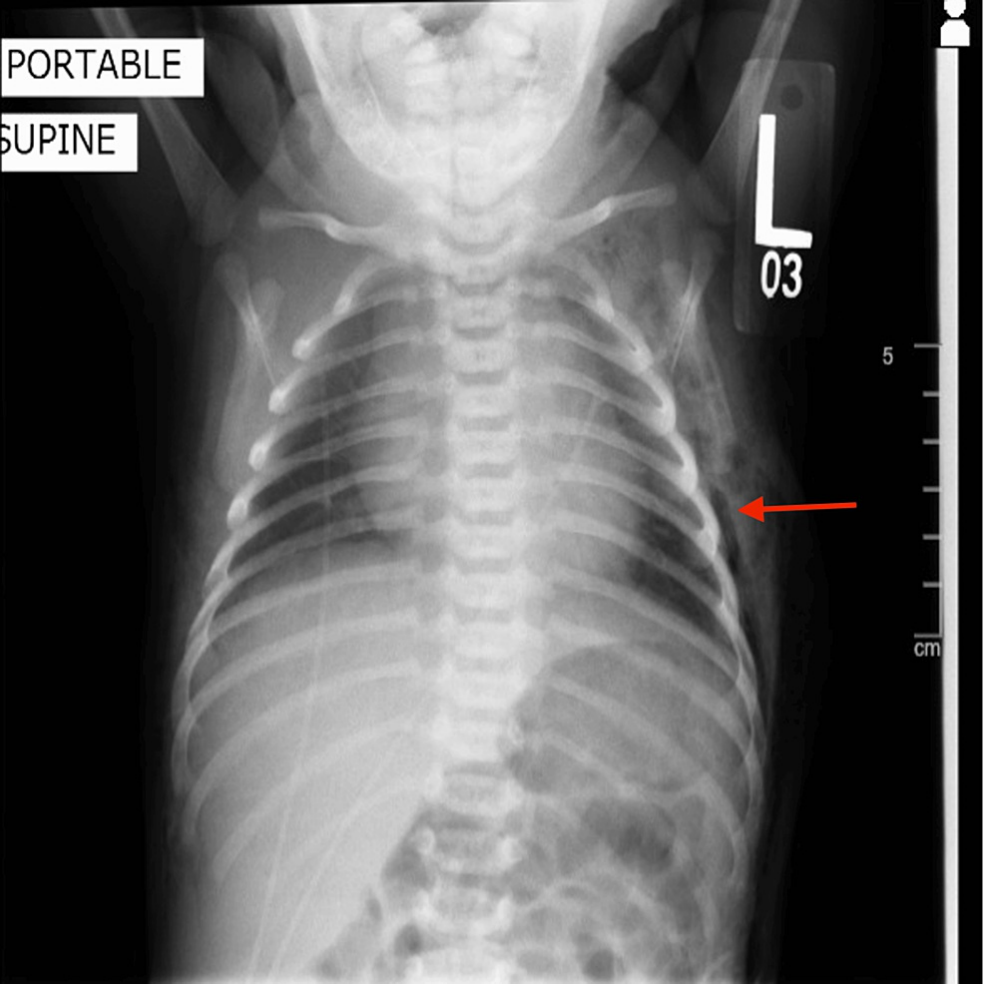
Chest X-ray on the sixth day of life showing resolution of pneumothoraces bilaterally with continued presence of left-sided subcutaneous emphysema (red arrow).

On day six, repeat CXR showed that the bilateral pneumothoraces were fully resolved (Figure 3). SE was still present on imaging and crepitations were appreciated on the examination as before. The neonate was otherwise stable with no signs of distress. At this time, the neonate was discharged home with plans for outpatient follow-up with their pediatrician.

## Discussion

Tube thoracostomy is one of the most common thoracic procedures performed in operating rooms, emergency rooms, and intensive care units to remove fluid or air from the pleural space. The tubes can vary in size, particularly width, and there are different methods for inserting the tube. In neonates, methods for insertion include the use of a trocar or with a guidewire or pigtail catheter via the Seldinger technique. When placing a tube using a trocar, the drain and trocar may be using excessive force [5]. In comparison, a pigtail catheter via the Seldinger technique involves a smaller bore tube with a guidewire and tract dilators, usually with ultrasound guidance [4,6]. As an invasive procedure, any method of tube placement carries a substantial risk of complications. Broadly, complications fall into two categories, namely, infectious or technical [4,7]. More specifically, technical complications can be due to tube insertion, tube position, tube removal, or tube malfunction [4,7]. Compared to the pigtail catheter, the use of a trocar is believed to be associated with a higher complication rate due to the rigid nature of the trocar and the excess force required [4-7]. The most reported complication by trocar is insertional complications with injury to intrathoracic or extrathoracic organs [4]. In adults, Mohan et al. concluded that the trocar technique should be avoided and instead blunt dissection with digital exploration, another insertion method, should be utilized [8]. Another study by Dural et al. concluded that a modified technique combining trocar use with digital exploration resulted in fewer complications than a pure trocar approach in adults [5]. In neonates, a retrospective study comparing pigtail catheter insertion via the modified Seldinger technique to trocar thoracostomy found that the trocar technique resulted in longer procedure times. However, there was no statistically significant difference in length of chest tube stay, hospital stay, or complication rate [9]. Tube malposition/malfunction was the most common complication but without any significant difference between the two groups and neither group reported the development of infection, laceration, hemothorax, SE, or need for surgical intervention [9]. While pigtail catheters are generally considered safer than insertion with trocars, a separate neonatal study suggested that complications from pigtail catheters, mainly lung perforation in the upper lobes, are more common than believed [10].

SE has a variety of etiologies and can occur spontaneously, often as a sequela of infections, trauma, mechanical ventilation, or as complications from surgical/procedural interventions such as chest tube insertions [1]. In cases of trauma or procedural complications, the development of SE is thought to be due to injury to the parietal pleura which allows for air passage into the pleural and subcutaneous tissues [1]. A study that followed cases of SE over 10 years demonstrated that the mean age of patients with SE was 53 ± 14.83 years, with 71% of cases being male [1]. However, SE in neonates is a rare complication, most frequently occurring secondary to pneumothoraces and pneumomediastina [11]. The incidence of pneumothorax in neonates is found to be 1% in term neonates and 6-10% in preterm neonates [12]. A pneumomediastinum is, like the word itself suggests, air trapped in the mediastinal area [1,13] and was similarly found to have a 2.3% incidence in term neonates, 2% in preterm neonates, and 1% in neonates born via cesarean section [14]. Spontaneous pneumothorax can result in SE via the Macklin effect where alveolar rupture is followed by air leaking into the loose connective tissue that surrounds the pulmonary vasculature. From there it tracks along the broncho-vascular sheath and is therein free to continue along fascial planes [15]. Although our patient had a spontaneous pneumothorax, signs of subacute emphysema only began to develop about 10 hours after tube thoracostomy using the trocar technique. This is suggestive that for our patient, SE was most likely a result of the placement of the chest tube rather than the original spontaneous pneumothorax.

In patients with chest tubes, regardless of insertion technique, SE can occur when the parietal pleura is breached, which then creates a pathway for air to directly enter the surrounding tissue. With the drain now in place, it is hypothesized that SE continues to develop due to the volume of air passing from the parietal pleura to the subcutaneous tissue exceeding the amount of air being removed from the pleural cavity via the drain [15].

For some patients, such as ours, rapid treatment is often preferred and necessary. Neonates have a flexible chest wall, frail lung tissue, and vital structures in proximity. As they have little compensatory lung reserve, in cases where they develop a pneumothorax, it can rapidly evolve into a serious situation [9]. For that reason, it is imperative that those in the NICU be prepared to diagnose and rapidly treat air leaks to avoid morbidity and mortality [9]. It is for that reason perhaps that the trocar technique is still used in NICU patients as some may feel they are more comfortable with the method. In our NICU, for example, the trocar technique was used because it was the method that most of the staff was trained and comfortable using. However, this advantage does not outweigh the high rate of complications associated with this technique, especially for already vulnerable patients such as neonates. In the case of our patient, the use of the trocar technique most likely directly led to the development of SE. SE is usually self-limiting and nonfatal but is not without its risks [1]. The expansion of air into the subcutaneous tissues can interrupt proper lung expansion which prevents patients from reaching appropriate tidal volumes. This can result in oxygen desaturation, respiratory distress, and even cardiac arrest [1]. Since our patient, the Seldinger technique with pigtail catheters has become the preferred method for chest tube placement.

Management of SE starts with the treatment of the underlying cause, which generally leads to gradual resolution of the emphysema. For mild cases, observation is indicated, as it is typically self-limiting and resolves in 10 days or less if the cause is treated [1]. In more severe cases, high-concentration oxygen (high FiO_2_) is generally recommended. Oxygen replaces nitrogen in the pneumothorax and allows for gaseous diffusion and resolution of the pneumothorax and SE [1]. In the case of our patient, despite having clinical signs of huge crepitus as a result of SE, the patient remained clinically stable and showed no signs of respiratory distress. This complication resolved spontaneously with respiratory and symptomatic management. SE, in rare cases, can progress to a life-threatening condition [1]. In our index case, the SE was resolving without treatment at the time the infant was discharged home, thus following the pattern of most SE cases which are typically self-limiting.

While data regarding risks and benefits are readily available for the adult population, data from the neonatal world tend to be scarce. We reported a case of a late-term neonate who underwent chest tube placement via the use of a trocar that resulted in SE to enrich the literature on this topic. Our paper is limited to just one case and research comparing the trocar technique to the Seldinger with pigtail catheter technique in our specific NICU has not taken place at this time. Further research regarding SE management and follow-up in the neonatal population is warranted. For example, as our patient had multiple CXRs during their stay, further research into the indications for CXR repetition and the possible use of point-of-care ultrasound in such cases may be helpful.

## Conclusions

SE is a rare complication of pneumothorax or chest tube placement, especially when using a trocar. Increased awareness of neonatal procedures leading to complications is highlighted in this case report. As some complications can be life-threatening, we respectfully advise neonatologists to consider all complications of procedures and report them, such as we have here, to enrich the data coming from the neonatology world.
